# Mediators of socioeconomic inequalities in preterm birth: a systematic review

**DOI:** 10.1186/s12889-022-13438-9

**Published:** 2022-06-07

**Authors:** Philip McHale, Gillian Maudsley, Andy Pennington, Daniela K. Schlüter, Ben Barr, Shantini Paranjothy, David Taylor-Robinson

**Affiliations:** 1 Department of Public Health, Policy and Systems, Institute of Population Health, University of Liverpool, Liverpool, England; 2 School of Medicine, Medical Sciences and Nutrition, Aberdeen Health Data Science Research Centre, University of Aberdeen, Aberdeen, Scotland

**Keywords:** Preterm birth, Mediation, Socioeconomic inequalities, Maternal smoking, Causal inference

## Abstract

**Background:**

Rates of preterm birth are substantial with significant inequalities. Understanding the role of risk factors on the pathway from maternal socioeconomic status (SES) to preterm birth can help inform interventions and policy. This study therefore aimed to identify mediators of the relationship between maternal SES and preterm birth, assess the strength of evidence, and evaluate the quality of methods used to assess mediation.

**Methods:**

Using Scopus, Medline OVID, “Medline In Process & Other Non-Indexed Citation”, PsycINFO, and Social Science Citation Index (via Web of Science), search terms combined variations on mediation, socioeconomic status, and preterm birth. Citation and advanced Google searches supplemented this. Inclusion criteria guided screening and selection of observational studies Jan-2000 to July-2020. The metric extracted was the proportion of socioeconomic inequality in preterm birth explained by each mediator (e.g. ‘proportion eliminated’). Included studies were narratively synthesised.

**Results:**

Of 22 studies included, over one-half used cohort design. Most studies had potential measurement bias for mediators, and only two studies fully adjusted for key confounders. Eighteen studies found significant socioeconomic inequalities in preterm birth. Studies assessed six groups of potential mediators: maternal smoking; maternal mental health; maternal physical health (including body mass index (BMI)); maternal lifestyle (including alcohol consumption); healthcare; and working and environmental conditions. There was high confidence of smoking during pregnancy (most frequently examined mediator) and maternal physical health mediating inequalities in preterm birth. Significant residual inequalities frequently remained. Difference-of-coefficients between models was the most common mediation analysis approach, only six studies assessed exposure-mediator interaction, and only two considered causal assumptions.

**Conclusions:**

The substantial socioeconomic inequalities in preterm birth are only partly explained by six groups of mediators that have been studied, particularly maternal smoking in pregnancy. There is, however, a large residual direct effect of SES evident in most studies. Despite the mediation analysis approaches used limiting our ability to make causal inference, these findings highlight potential ways of intervening to reduce such inequalities. A focus on modifiable socioeconomic determinants, such as reducing poverty and educational inequality, is probably necessary to address inequalities in preterm birth, alongside action on mediating pathways.

**Supplementary Information:**

The online version contains supplementary material available at 10.1186/s12889-022-13438-9.

## Background

Preterm birth, defined as birth before 37 weeks’ gestation, is a substantial public health problem, accounting for nearly 11% of births globally. Prevalence varies across regions and is increasing in most countries [1]. Inequalities on the basis of various individual and area level measures of maternal socioeconomic status (SES) are consistently demonstrated [2], with estimates from Europe indicating an almost 50% higher prevalence among the least compared with most educated mothers [3, 4], and a substantial proportion of negative perinatal outcomes is attributed to socioeconomic inequalities [5].

Preterm birth has serious negative health, educational, and social outcomes [6] and is a leading cause of mortality in children under five. Therefore, understanding how to reduce inequalities in preterm birth represents a clear policy aim for reducing health inequalities more broadly. For example, studies have shown that preterm birth is an important driver of inequalities in child mortality, mental health, asthma and obesity [1, 7, 8].

Studies of socioeconomic inequalities in preterm birth have indicated that maternal factors on the causal pathway from maternal SES to preterm birth may partly explain inequalities, however the impact of these factors is unclear [9]. These intermediate maternal factors, or mediators, include known risks for preterm birth: smoking during pregnancy, low or high body mass index (BMI), and poor pre-pregnancy maternal health [10–12]. These risks, and other health system factors, such as access to antenatal care, are potential contributors to differences in preterm birth between groups [13] and are socially patterned.

A potentially effective way to reduce inequalities in preterm birth is through intervention on mediating pathways linking maternal SES and risk of preterm birth. Mediation is the mechanism whereby an exposure affects an outcome indirectly through a third variable that sits on the causal pathway from exposure to outcome. There has been rapid development of methods to assess mediation in observational data over the last ten years. These methods have increased our ability to make causal interpretations under specific assumptions, using the counterfactual framework [14]. The assumptions are that: a) there is no unmeasured confounding of exposure-outcome, exposure-mediator, and mediator-outcome pathways and b) no confounder of the mediator-outcome pathway is also caused by the exposure (‘cross-world independence’).

The evidence for mediation of socioeconomic inequalities in preterm birth has not, however, been systematically assessed in the context of these new advances. This review therefore aims to identify mediators of the relationship between maternal SES and preterm birth, assess the strength of evidence, and evaluate the quality of methods used to assess mediation.

## Methods

This review sought empirical studies published between January 2000 and July 2020 that address the research question: ‘How do key risk factors, such as maternal health, maternal behaviours, and system-level factors, mediate the effect of maternal socioeconomic status on preterm birth?’. The protocol was registered with the Prospective Register for Systematic Reviews (PROSPERO) (Registration code: PROSPERO 2020 CRD42020203613). Ethics approval was not required. Reporting complies with Preferred Reporting Items for Systematic Reviews and Meta-Analyses (PRISMA) guidance (as per PRISMA statement, Additional file 1). Minor deviations from the PROSPERO protocol (as detailed in Additional file 2) have not impacted on our findings or introduced a new risk of bias.

### Search strategy

Searches used five databases: Scopus, Medline OVID, “Medline In Process & Other Non-Indexed Citation”, PsycINFO and Social Science Citation Index (via Web of Science). Search terms were informed by an existing systematic review for mediation [15] and followed the PICO structure (Table 1). Searches were supplemented using the same search terms through Advanced Google Searches. Search terms combined variations on mediation, SES, and preterm birth (Additional file 3).

**Table 1:** Inclusion/Exclusion criteria for systematic review

	**Include**	**Exclude**
Population	Pregnant women	
Intervention / mediator	Behavioural risk factors (e.g. smoking, alcohol). Social risk factors (e.g. Environmental (housing, working)). Maternal health status (both mental and physical health)	Genetic risks for preterm birth
Comparison across exposure	Comparison across socioeconomic strata (either individual or area-based)	
Outcomes	Preterm birth and gestational age	Other birth outcomes (e.g. low birthweight)
**Publication characteristics: Inclusion / exclusion criteria**		
	**Include**	**Exclude**
Publication types	Primary studies from peer-reviewed literature, including those from reviews. Relevant secondary analyses (meta-analysis). Papers published or in-press. Working papers	Not primary research, e.g. letters, editorials, commentaries, conference proceedings, books and book chapters, meeting abstracts, lectures, and addresses. Previous reviews and meta-analyses, but relevant reviews were used to identify relevant primary studies
Types of study	Analytical techniques that are relevant to research question: --Mediation --Attenuation Differential exposure	Other methods. Mediation or attenuation not specifically calculated within analysis
Year of publication	2000–2020	
Language	English language	

Different measures of SES (e.g. parental education, occupation, income and neighbourhood factors) were all included. Maternal SES can be used to measure inequalities in broadly two ways; individual or area-based measures. Individual measures include educational attainment, income, and occupation, and may be further classified as measures for the mother and for the household (e.g. for income). Area-based measures can include census tracts or composite scores for deprivation and are frequently used as a proxy measure for individual SES.

The starting time period cut-off of 2000 was used as the focus of the review was on the application of recent advancements in mediation analysis techniques to the evidence base. Therefore, studies before 2000 would not be relevant.

All included studies were hand-searched for backward citations (using reference lists) and forward citations (using Web of Science). Studies included in relevant systematic reviews identified were also assessed [16–23]. Screening used EPPI-Reviewer 4 systematic review management software [24].

### Selection

On screening titles and abstracts, those mentioning mediation or explanation of inequalities in preterm birth were then reviewed against the inclusion/exclusion criteria (Table 1). Approximately 15% of titles-abstracts were dual-screened and calibrated to ensure consistent screening. The remaining titles-abstracts were single-screened. Included papers underwent full-text screening independently by two reviewers. A third reviewer was available to settle remaining disagreements but was not needed. All study designs were included.

### Data extraction

All data were dual-extracted independently by two reviewers. Data extracted included study design, population, time period, sample size, measure of maternal SES, mediators examined, mediation analysis approach, total effect of SES on preterm birth, indirect effect through the mediator, significance of pathways, and proportion eliminated through mediation (the standardised metric used in synthesis, Table 2) [25]. For studies not providing proportion eliminated, it was estimated by dividing the indirect effect via the mediator by the total effect. Significance of mediation was assessed using the indirect effect confidence intervals (CI) primarily, if available, or the p value of the effect. Proportion eliminated was selected to synthesise the range and distribution of mediated effects [25]. Meta-analysis was not appropriate because the mediators investigated and methods of calculating mediation effects differed between studies, and many studies lacked significance estimates.

**Table 2:** Description of mediation effects

Effect Measure	Description
Total Effect	The overall effect of the exposure on an outcome: --For the difference method, this is the regression output for the exposure when not adjusted for the mediator. --For product of coefficients, this is the sum of direct and indirect effect
Direct Effect	The effect of the exposure on an outcome when the intermediate variable is removed
Indirect Effect	The effect of the exposure on an outcome through an intermediate variable
Proportion Eliminated	How much of the total effect would be removed through action on the intermediate variable (setting the mediator to the same level for all pregnant women) [26]: --For the difference method, this is the difference between the total effect and regression output for the mediator-adjusted regression, divided by total effect (minus one if using exponentiated outputs) --For product of coefficients, this is the indirect effect divided by total effect [14]

### Quality-scoring

Studies were quality-assessed using a hybrid approach. This assessed study quality through the risk of bias and the quality of the mediation methods used (Additional file 4). Risk of bias associated with study design was assessed using the Liverpool University Quality Assessment Tool relevant to the particular study design [27]. Given that there is no standard approach for quality assessment of mediation analyses, we added three criteria based on a previous mediation review and on qualitative work informing reporting guidelines for studies of mediation [15, 28]. Aspects of study design relevant to mediation analysis included: consideration of exposure-mediator interaction in the analysis; a directed acyclic graph (DAG) [29] informing the mediation analysis; and consideration of causal assumptions underpinning the mediation analysis.

### Integration

Studies were synthesised narratively, and results were grouped by mediator. The order of reporting results in text was based upon frequency of the mediator in the included studies and the quality-scoring [30, 31]. The certainty of the evidence for each mediator was assessed by considering the sample size, quality score, and consistency of the direction of mediated effects (GRADE). Criteria for publication bias and imprecision could not be calculated [25]. A harvest plot displayed the range of proportion eliminated for the four most studied mediators [32]. Results from the review were then used to identify mediators and confounders, which were integrated into a DAG [29].

## Results

### Search results and description of included studies

**Fig. 1:**
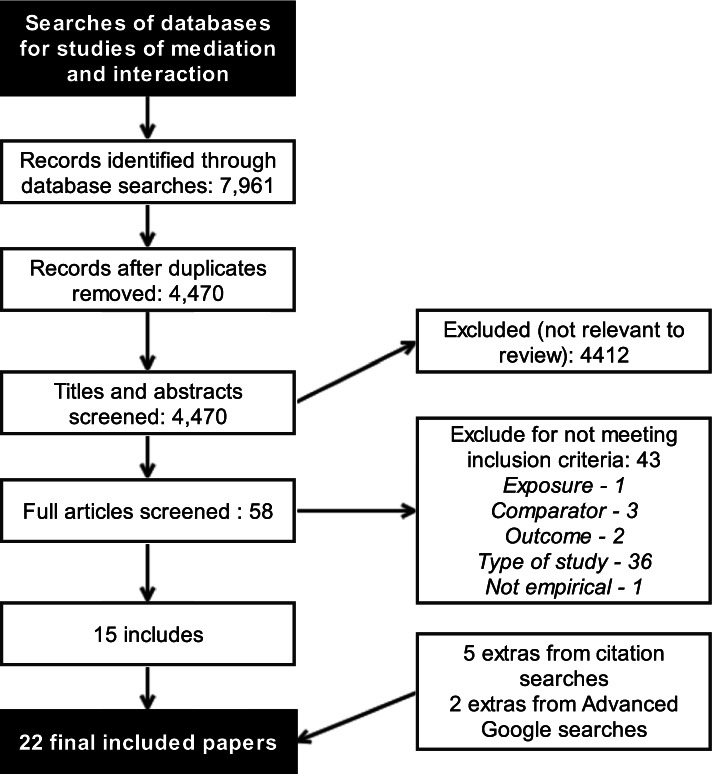
PRISMA diagram for included studies for the systematic review question

After removing duplicates, the initial searches identified 4,470 papers to review, of which 58 were full-text screened (Fig. 1). After screening and citation searches, 22 studies were included [33–54]. Over half of the studies used cohort design. Ten were from Europe (all North and West Europe) [33, 43, 46–53], eight were from North America (six from USA, two from Canada) [34, 36–39, 41, 42, 44], two from Iran [35, 40], and one each from Ghana [45] and Brazil (Table 3) [54]. One study did not specify the study period, and the other 21 covered periods between 1980 and 2013. Another excluded study did not quantify results for the mediation of the SES effect on preterm birth by smoking [55]. Only one study provided the CI for the proportion eliminated [33].

**Table 3:** Characteristics of included studies (*n* = 22) about mediation between socioeconomic status (SES) and preterm birth

Paper	Design	Country	Sample Size and Characteristics	Study Period	Mediation Analysis Approach	Measure of SES	Quality Score (/13)
Poulsen et al. (2019) [33]	Cohort	Denmark	77,020 – National birth cohort (whole)	NS	Difference method using risk differences from linear regression	Maternal education: Short (≤ lower secondary) to long (degree; reference)	10
		Netherlands	4,508 – Rotterdam birth cohort (whole)	NS			
		Norway	78,267 – National birth cohort (whole)	NS			
Ross et al. (2019) [34]	Cohort	United States (US)	718,952 –Californian birth cohort (whole)	2007–2012	Product of coefficients/ Path analysis using Lavaan Package	Maternal education: At most high-school to more than high school (reference)	9
Dolatian et al. (2014) [35]	Cohort	Iran	500 – Random sample of pregnant women from stratified sample of four Tehran hospitals	2011–2012	Product of coefficients/ Path analysis using Lisrel Software	Income	9
Clayborne et al. (2017) [36]	Cohort	Canada	2,068 – Sample of pregnant women from Calgary and Edmonton Metropolitan Regions	2008–2012	Product of coefficients using PROCESS macro	Neighbourhood SES	8
Dooley (2009) [37] [PhD thesis]	Cross-sectional	US	28,793 – Hamilton County, Ohio, birth cohort (whole)	2001–2003	Product of coefficients/ Path analysis of multilevel modelling using Mplus	Neighbourhood concentrated disadvantage	8
Mehra et al. (2019) [38]	Cohort	US	138,494 – National convenience sample (retrospective) of births from all states using health insurance data	2011	Product of coefficients/ Path analysis of multilevel modelling using Mplus	Neighbourhood SES: most deprived quarter to least deprived (reference)	8
Meng et al. (2013) [39]	Cross-sectional	Canada	90,500—All births (including multiple) at three Ontario province public health units	2000–2008	Product of coefficients of multilevel modelling using both linear and logistic regression	Neighbourhood SES	8
Mirabzadeh et al. (2013) [40]	Cohort	Iran	500 – Random sample of pregnant women from stratified sample of four Tehran hospitals	2012–2013	Product of coefficients/ Path analysis using Lisrel Software	Composite comprising: maternal and spousal education, persons and cost/household area, car, computer	8
Misra et al. (2001) ^a^[41]	Cross-sectional	US	735 – Urban university hospital sample of births to black mothers: drug users, women without prenatal care, and a systematic sample of the rest	1995–1996	Difference method using logistic regression	Lack of time and money	8
Nkansah-Amankra et al. (2010) [42]	Cross-sectional	US	8,064 – South Carolina state, stratified systematic sample of births	2000–2003	Difference method using multilevel logistic modelling	Neighbourhood SES: Proportion of residents in poverty	8
Räisänen et al. (2013) [43]	Cross-sectional^b^	Finland	1,390,742 – National birth cohort (whole)	1987–2010	Difference method using logistic regression	Maternal occupation; blue collar relative to upper white collar (reference)	8
Ahern et al. (2003) [44]	Case–Control	US	1,496 cases + controls – A San Francisco hospital based sample of births: All preterm plus random selections of full-term, stratified by African American and White	1980–1990	Difference method using multilevel logistic modelling	Neighbourhood context	7
Amegah et al. (2013) [45]	Cross-sectional	Ghana	559 – Cape Coast’s four main healthcare facilities, random sample weighted by hospital or urban centre	2011	Difference method: Generalised linear model using Poisson Distribution and log link	Level of monthly income: low to upper middle and high (reference)	7
van den Berg et al. (2012) [46]	Cohort	Netherlands	3,821 – Amsterdam birth cohort (Dutch-only) (whole)	2003–2004	Difference method using logistic regression	Maternal education: years of education after primary school, low (< 6) to high (> 10; reference)	7
Morgen et al. (2008) [47]	Cohort	Denmark	38,131 primiparous & 37,849 multiparous – National birth cohort	1996–2002	Difference method using Cox regression	Maternal education; < 10 years to > 12 years (reference)	7
Gisselmann and Hemström (2008) [48]	Cross-sectional	Sweden	356,887 – National birth cohort (whole)	1980–1985	Difference method using logistic regression	Maternal occupation: Unskilled manufacturing manuals to middle non-manuals (reference)	7
Niedhammer et al. (2012) [49]	Cohort	Republic of Ireland	913 – Random sample of pregnant women (Irish-only) from two hospitals (urban and rural)	2001–2003	Difference method using Cox Regression	Maternal education: lower than to higher than secondary (reference)	7
Jansen et al. (2009) [50]	Cohort	Netherlands	3,830 – Rotterdam birth cohort (whole)	2002–2006	Difference method using logistic regression	Maternal education: low (< 4 years general secondary) to high (Master degree, PhD; reference)	7
Quispel et al. (2014) ^a^[51]	Cohort	Netherlands	1,013 – Rotterdam, Apeldoorn, Breda: Random samples of pregnant women from primary, secondary, tertiary care	2009–2011	Difference method using logistic regression	Maternal education: low to moderate (reference)	6
Gissler et al. (2003) [52]	Cross-sectional	Finland	548,913 – National birth cohort (whole)	1991–1999	Difference method using logistic regression	Maternal occupation: blue collar to upper white collar (reference)	6
Gray et al. (2008) [53]	Cohort	Scotland	400,752 – National (hospital) birth cohort (whole)	1994–2003	Difference method using logistic regression	Neighbourhood SES: most deprived fifth to least deprived (area-based) (reference)	6
de Oliveira et al. (2019) [54]	Case–Control	Brazil	296 cases + 329 controls – Londrina sample of hospital births (including multiple)	2006–2007	Structural equation modelling	Socioeconomic vulnerability	4

*NS* not stated

^a^ Not specified if Misra et al. (2001) [41] and Quispel et al. (2014) [51] excluded multiple births. Meng et al. (2013) [39] and de Oliveira et al. (2019) [54] included multiple births. All other studies excluded multiple births

^b^ despite being labelled as a case–control study

Ordered by Quality Score

### Quality assessment

Additional file 5 shows the quality-scoring for each study. In all the cohort studies there was risk of either selection bias (4/12) [34, 36, 38, 46], response bias (6/12) [35, 40, 49–51, 53], or bias in follow-up (3/12) [33, 47, 51]. Of the two case-control studies, one had a risk of bias in selection of both cases and controls [54]. Of the cross-sectional studies, three showed low risk of bias, but the others showed potential selection bias (1/8) [52] and response bias (4/8) [37, 39, 41, 45].

Fifteen studies used individual measures of maternal SES, and seven used aggregated measures (e.g. neighbourhood SES) [36–39, 42, 44, 53]. Potential measurement bias for the mediators featured in 14 studies (mostly from self-reported smoking) [33, 37, 39, 42–50, 52, 53], while nine explained measurement of preterm birth inadequately [34, 36, 38, 39, 44, 48, 51, 52, 54]. Of the three confounders identified (maternal age, parity, and race or ethnicity – see below), three studies adjusted for none [40, 51, 54], 17 adjusted for one or two [33, 35, 37–39, 41–50, 52, 53], and two adjusted for all three variables [34, 36].

### Mediation approach

The ‘difference method’ was the most frequently used approach to assess mediation (14 studies) [33, 41–53], estimating the ‘controlled direct effect’ [14]. Other approaches used product of coefficients (seven, with path analysis in five) [34–40] and, in one, structural equation modelling not specified as path analysis [54]. Only one of the studies using the difference method estimated the statistical significance of the mediating effect, using bootstrapping to estimate CI [33].

Regarding quality of mediation analysis: 11 studies included graphical representation (DAG) of the mediated pathway [33–41, 51, 54], six studies examined exposure-mediator interaction in their analysis [33, 37–39, 42, 44], and only two studies explicitly considered the causal assumptions; these studies included all three of these quality indicators [33, 39]. The temporal nature of measurement of exposure and outcome was unclear in eight studies[33, 34, 36, 37, 48, 52–54]. They were measured synchronously in four studies,[39, 41, 43, 45] and measurement of the exposure preceded that of the outcome in nine studies [35, 38, 40, 42, 46, 47, 49–51]. In one study, the exposure measures were from census data collected 1980–1990, the same time period as the outcome [44].

### Association of SES and preterm birth

Ten different measures of maternal SES were used, broadly either individual or area-based (Table 3). Six separate individual level measures were used; maternal education was the most frequent (*n* = 7) [33, 34, 46, 47, 49–51], followed by occupation (each *n* = 3) [43, 48, 52], two used income [35, 45], two used different composite measures [40, 54], and one used perceived lack of time and money [41]. Four measures were area-based: a composite SES score (*n* = 4) [36, 38, 39, 53], the proportion of residents in poverty [42], a measure of disadvantage [37], and measures of neighbourhood context (for African-American mothers: median income and proportion of adult male unemployment in 1990; for white women: change in proportion of adult male unemployment 1980–1990) [44].

Eighteen studies found that lower SES was significantly associated with increased preterm birth, using both individual and area-based measures (Table 4). Three found no significant association [36, 42, 54], while one found an association for African-American participants only [44]. Two of the studies finding no significant association measured the effect of neighbourhood SES while controlling for individual measures of SES.

**Table 4:** Estimated total effect and standardised mediator effect (proportion eliminated by mediation) from included studies (*n* = 22)

Paper	Effect of SES on PTB (95% confidence interval if available)	Mediator	Prevalence of mediator in sample	Proportion eliminated (95% confidence interval if available)
Poulsen et al. (2019) [33] Denmark	Total effect RD: 2.0 (1.4, 2.5) excess PTB/100 singleton deliveries	Smoking	17% total; 39% short education, 8% long	22% (11%, 31%)^a^
Poulsen et al. (2019) [33] Netherlands	Total effect RD: 3.2 (0.8, 5.2)		19% total; 41% short education, 7% long	10% (-22%, 29%)
Poulsen et al. (2019) [33] Norway	Total effect RD: 2.0 (0.9, 3.0)		9% total; 34% short education, 4% long	19% (-1%, 29%)^a^
Ross et al. (2019) [34]	Direct coefficient: 0.072^*^ Total effect coefficient 0.077	Pre-eclampsia	5% in black women, 3% in white women	6.5%^a^
Dolatian et al. (2014) [35]	Direct coefficient: 0.06^*^ Total effect coefficient: 0.06126^*^	Perceived stress	Mean	11.8%^a^
		Perceived social support through stress	Mean	Mediated effect in opposite direction^a^
		Combined		2.1%^a^
Clayborne et al. (2017) [36]	Total effect OR: 0.91 (0.64, 1.31)	Pre-pregnancy body mass index (BMI)	Mean	Cannot be estimated
		Gestational weight gain	Mean	Cannot be estimated
		Combined		Cannot be estimated^a^
Dooley (2009) [37] (PhD thesis)	Direct effect: 43.29% increase in odds/standard deviation increase. Total effect^**^: 46.01%^*^	Medical risk	13%	2.9%^a^
		Smoking	13%	3.0%^a^
		Perceived neighbourhood support	Mean	No indirect effect
Mehra et al. (2019) [38]	Direct effect coefficient: 0.036. Total effect coefficient: 0.059^*^	Hypertension	10%	22.0%^a^
		Infection	28%	16.9%^a^
Meng et al. (2013) [39]	Total effect coefficient: 0.981 (0.626–1.337)	SES-related support	Composite measure	11.7%^a^
		Psychosocial	Composite measure	2.1%^a^
		Behavioural	Composite measure	5.5%^a^
		Health	Composite measure	6.4%^a^
Mirabzadeh et al. (2013) [40]	Total effect coefficient: 0.1441a	Perceived social support through stress	Mean	8.1%^a^
		Stress, depression, and anxiety	Mean	22.5%^a^
		Combined		30.6%^a^
Misra et al. (2001) [41]	Total effect OR: 2.85 (1.85–4.40)	Psychosocial factors only	26% severe stress	44%
		Biomedical and psychosocial factors	5% chronic disease	64%
Nkansah-Amankra et al. (2010) [42]	Total effect OR 1.34 (0.80–2.25)	Maternal stress (emotional, financial, spousal-related, traumatic)	14% low poverty, 57% high poverty	No significant total effect
Räisänen et al. (2013) [43]	Total effect OR: Extremely PTB 1.61 (1.38–1.89); Very PTB 1.48 (1.31–1.68); Moderately PTB 1.27 (1.22–1.32)	Smoking	12% to 18% by gestational age category	26% for extremely PTB 33% for very PTB 30% for moderately PTB
		Other factors and smoking	Composite measure	39% for extremely PTB 50% for very PTB 41% for moderately PTB
Ahern et al. (2003) [44] African-American	Total effect parameter estimate proportion unemployed: 44.4^*^	Cigarettes per day	Mean	3%
Ahern et al. (2003) [44] White	Total effect parameter estimate change in unemployed: -3.32			No significant total effect
Amegah et al. (2013) [45]	Total effect RR: 1.83 (1.31–2.56)	Malaria infection during pregnancy	48%	No effect
		Pre-pregnancy BMI	33% healthy weight	17%
		Cooking fuel used	18% LPG, 24% charcoal, 5% firewood	22%
		Combined		30%
van den Berg et al. (2012) [46]	Total effect OR: 1.95 (1.19–3.20)	Smoking	7% total, 33% in low educated, 2% in high educated	43%
		Smoking and environmental tobacco exposure	6% total, 27% in low educated, 1% in high educated	39%
Morgen et al. (2008) [47]	HR primiparous: 1.22 (1.04–1.42) HR multiparous: 1.56 (1.31–1.87)	Smoking	26% to 35% by gestational age category	5% in primiparous 23% in multiparous
		Alcohol	40% to 45% by gestational age category	5% in primiparous 4% in multiparous
		Binge drinking	25% to 26% by gestational age category	5% in primiparous no effect in multiparous
		Pre-pregnancy BMI	Mean	9% in primiparous 2% in multiparous
		Gestational weight gain	Mean	5% in primiparous 4% in multiparous
		Combined		23% in primiparous 30% in multiparous
Gisselmann and Hemström (2008) [48]	Total effect OR: 1.41^*^	Job control	Not stated	44%
		Job hazards	Not stated	5%
		Physical demands	Not stated	22%
		All working conditions	Not stated	46%
Niedhammer et al. (2012) [49]	Total effect HR: 2.14 (1.05–4.38)	Rented home	43% lower than secondary, 15% higher than secondary	26%
		Crowded home	18% lower than secondary, 5% higher than secondary	13%
		Material factors	Composite	33%
		Smoking	46% lower than secondary, 16% higher than secondary	2%
		Alcohol	50% lower than secondary, 62% higher than secondary	14%
		Behavioural	Composite	10%
		Saturated fatty acids (nutritional factors)	31% lower than secondary, 20% higher than secondary	14%
		Material + behavioural	Composite Measure	38%
		Material + behavioural + nutritional	Composite Measure	42%
Jansen et al. (2009) [50]	Total effect OR: 1.89 (1.28–2.80)	Mother’s age	Mean	22%
		Mothers’ height	Mean	22%
		Preeclampsia	2% total, 1% high, 4% low education	13%
		Intrauterine growth restriction (IUGR)	1% total, 1% high, 2% low education	12%
		Marital status (single)	8% total, 3% high, 20% low education	2%
		Pregnancy planning (unplanned)	19% total, 10% high, 34% low education	No effect
		Financial concerns	12% total, 5% high, 30% low education	19%
		Long-lasting difficulties	Mean	11%
		Psychopathology	Mean	16%
		Working hours	Mean	No effect
		Smoking	18% total, 5% high, 45% low education	8%
		Alcohol consumption	50% total, 68% high, 25% low education	17%
		BMI	67% total healthy weight, 75% high, 51% low education	7%
		All except preeclampsia/IUGR/ working hours/pregnancy planning	Composite Measure	69%
		All except working hours/pregnancy planning	Composite Measure	89%
Quispel et al. (2014) [51]	Total effect OR: 1.06 (1.02–1.10)	Depression score	15%	No effect
Gissler et al. (2003) [52]	Total effect OR: 1.35 (1.25–1.45)	Smoking	15% total, 5% upper white collar, 26% blue collar workers	42%
Gray et al. (2008) [53]	Total effect OR: 1.49 (1.43–1.54)	Smoking	For 2 periods: 30% & 29% total, 15% for both periods in least deprived, 43% & 39% in most deprived	45%
de Oliveira et al. (2019) [54]	Direct standardised estimate: -0.083	Inadequate prenatal care	Not stated	Cannot be estimated^a^
		Unwanted pregnancy		Cannot be estimated^a^

^*^ *p* value < 0.05, ^**^ Percentage change in the odds per standard deviation increase

^a^ indirect effect significant, *PTB* preterm birth, *HR* hazard ratio, *OR* odds ratio, *RR* relative risk, *RD* risk difference, coefficient = beta coefficient, *LPG* liquefied petroleum gas, *Mean* mean score so prevalence score not calculable

### Mediators

**Fig. 2:**
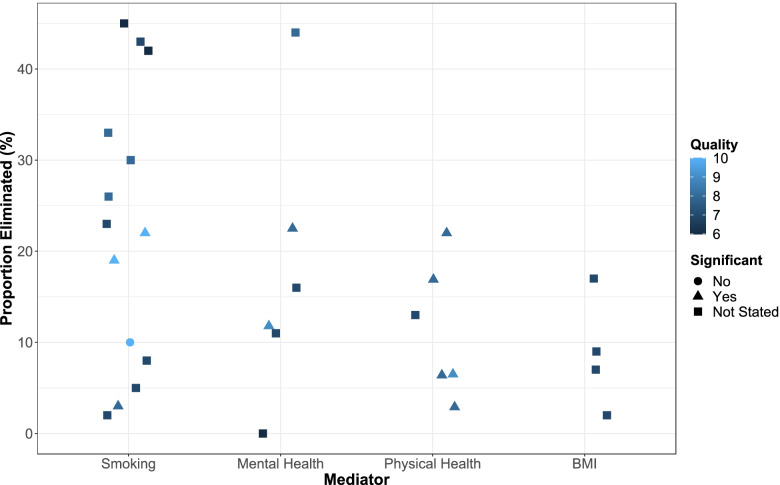
Harvest plot of proportion eliminated metric for the four most commonly examined mediators. Proportion eliminated: proportion that differences in preterm birth between socioeconomic groups would be reduced by if the mediator was the same for all pregnant women. Colour shows quality score (lighter shade indicates higher score) and shape is significance of indirect effect. Only studies with a significant total effect of SES on preterm birth were included and a study using a continuous measure of smoking was not included. *BMI* body mass index

The most assessed mediators by the ‘proportion eliminated’ metric were: maternal smoking during pregnancy; mental health; physical health conditions; and BMI (Fig. 2).

### Maternal smoking during pregnancy

Ten studies reported the potential mediating effect of smoking, the most frequent mediator studied, with sixteen estimates of proportion eliminated metric. One of these studies used number of cigarettes smoked [44], two categorised number smoked (none, 1–10, more than 10 cigarettes) [33, 47], two included an ex-smoking category [49, 50], one used a mixture of binary variable (yes/no) and the addition of quitters for later in the study period [43], and four used binary variables (smoker/non-smoker) only [37, 46, 52, 53]. Two estimates used cigarettes smoked as a linear variable (thus excluded from Fig. 2 as not comparable to categorised results).

The 16 estimates ranged from 2% eliminated [49] to 45% [53]. Only two studies reported the significance of the indirect effect. Poulsen et al. [33] estimated a significant indirect effect in Denmark and Norway, equating to a significant proportion eliminated of 22% (95% CI 11%, 31%) in Denmark and non-significant proportion eliminated of 19% (-1%, 29%) in Norway. The same study also found a non-significant indirect effect through smoking in the Netherlands, where proportion eliminated was 10% (-22%, 29%), however there was a much smaller sample size. Dooley (2009) [37] found there was a significant indirect effect, equating to 3% eliminated (CIs not provided).

Räisänen et al. [43] reported the largest study (nearly 1.4 million births), finding the proportion eliminated was 26% for extremely preterm births (< 28 weeks gestation), 33% for very preterm births (28–32 weeks gestation), and 30% for moderately preterm births (32–37 weeks).

Ahern et al. [44] found that number of cigarettes smoked eliminated 3% of the SES effect on preterm birth in African American mothers while the SES effect in white mothers was not significant. Niedhammer et al. [49] found the proportion eliminated was 2%, and Jansen et al. [50] found that the proportion eliminated was 8%. These three studies had small sample sizes when compared with the other studies (all less than 4,000 participants). Another smaller study, van den Berg et al., found the proportion eliminated to be 43% [46].

One study of approximately 38,000 primiparous women found the proportion eliminated was 5% [47]. Notably, the same study found the proportion eliminated was 23% in a similar number of multiparous women. Two large, lower quality studies (*n* = 400,752 and *n* = 548,913) found proportion eliminated was over 40% [52, 53].

### Maternal mental health

Six studies assessed the potential indirect effect of SES on preterm birth via maternal mental health. All studies used verified scales, with two focused on stress, depression, or anxiety measured during pregnancy [35, 40], two focused on stress alone (one measured during and one after) [41, 42], one focused on depression post-delivery [51], and one used both ‘general distress and psychiatric symptoms’ and stress one year pre-pregnancy [50]. Two studies also included assessment of level of social support and reported no direct effect on preterm birth [35, 40], which corresponded with Dooley finding no indirect effect of SES on preterm birth through support [37].

The six estimates of the proportion eliminated of the SES effect through maternal mental health ranged from 0 to 44%. Two studies estimated the significance of the indirect pathway, both finding significant indirect paths. Dolatian et al. [35] found that increased income apparently reduced stress and, maybe counterintuitively, perceived social support; increased stress was associated with reduced gestational age, while perceived social support increased gestational age by reducing stress. The proportion eliminated was 12% for stress alone, which reduced to 2% when support was also included. Notably, there was a discrepancy between the graphical results in the path model and the tabulated effects. Mirabzadeh et al. [40] found that the proportion eliminated for stress, depression, and anxiety was 22% and, when combined with level of social support, 31%.

None of the other studies estimated the significance of the indirect effect. Misra et al. [41] found that the proportion eliminated was 44% in black mothers. Nkansah-Amankra et al. [42] found the effect of SES on preterm birth was not significant prior to adjustment, therefore proportion eliminated is not an appropriate metric. Jansen et al. [50] found the proportion eliminated for psychopathology (measured using the Brief Symptom Inventory) was 16%, and for long-lasting difficulties (measured using questionnaire and interview in the year before pregnancy) was 11%. Quispel et al. [51] found there was no proportion eliminated. Mehra et al. [38] found there were no significant indirect effects through mental health conditions so was not reported.

### Maternal physical health

Six studies examined the potential mediation of the effect of SES on preterm birth through maternal physical health. Two studies examined pre-eclampsia [34, 50], three used composite measures to determine health (any health condition or one of a selection) [37, 39, 41], and one used specific medical conditions (hypertension and infection) [38]. The proportion eliminated ranged from 3 to 22% for physical health (Fig. 2, however this excludes the results for one of the composite measures).

Of the two studies that examined pre-eclampsia, one found a significant indirect effect while the other did not. Ross et al.  [34] found that the proportion eliminated was 6%. Notably, when the analysis was stratified for race, the effect of education on pre-eclampsia was less in black mothers and the indirect effect was smaller and no longer statistically significant. Jansen et al. [50] found that the proportion eliminated was 13% for pre-eclampsia.

Of the four studies that examined pre-existing health, three found significant indirect effects. Dooley (2009) [37] found that the proportion eliminated was 3% (maternal health conditions recorded on the birth certificate). Mehra et al. [38] found the proportion eliminated for hypertension was 22% and for infection was 17%. They found no significant indirect effects through diabetes mellitus so this was not reported. Meng et al. [39] found the proportion eliminated for an unspecified composite of maternal health challenges was 6%. Misra et al. [41] found that the addition of biomedical factors (chronic disease, vaginal bleeding, and no prenatal care) to psychosocial stress increased the proportion eliminated from 44 to 64%.

### BMI and gestational weight gain

Four studies measured mediation through pre-pregnancy BMI, with two also examining gestational weight gain. Only one study estimated whether the indirect effect was statistically significant. Clayborne et al. [36] found there was a significant indirect effect through BMI and gestational weight gain together but not separately. The proportion eliminated could not be calculated from the data provided.

The other three studies did not estimate statistical significance of the proportion eliminated or indirect effect. Amegah et al. [45] found that the proportion eliminated for BMI was 17%. Morgen et al. [47] found that the proportion eliminated for BMI was 9% and 2%, and for gestational weight gain was 5% and 4%, in primiparous and multiparous women, respectively. Jansen et al. [50] found the proportion eliminated was 7%.

### Maternal alcohol consumption in pregnancy

Three studies considered the mediating effect of categories of maternal alcohol consumption. Morgen et al. [47] found that for alcohol the proportion eliminated was 5% and 4% in primiparous and multiparous women, respectively. For binge drinking the proportion eliminated was 5% in primiparous women with no effect in multiparous women.

Niedhammer et al. [49] found the proportion eliminated was 14%. Jansen et al. [50] found the proportion eliminated 17%. None of the studies estimated statistical significance of the indirect effect. Notably, the two studies that reported prevalence of alcohol consumption by SES groups showed that consumption was more prevalent in higher than lower SES groups.

### Working and environmental conditions

Two studies examined environmental conditions. Amegah et al. [45] found the proportion eliminated for cooking fuel (as a measure of indoor air pollution) was 22%. van den Berg et al. [46] found the proportion eliminated for environmental tobacco exposure combined with cigarette-smoking was 39%, however the proportion eliminated was lower than for smoking alone (43%). Living conditions were examined, finding that the proportion eliminated for rented accommodation was 26% and for crowded housing was 13% [49].

Two studies examined working conditions. Gisselmann and Hemström (2008) [48] applied an aggregated measure of working exposure based on occupation, measured up to five years pre-birth. Proportion eliminated was: 46% for working conditions, 44% for job control, 22% for physical demands, and 5% for job hazards. These estimates were larger when analysis was limited to extremely preterm births. Jansen et al. [50] found working hours (measured in late pregnancy) had no indirect effect.

### Healthcare (antenatal care and family planning)

de OIliveira et al. found there were significant indirect effects through inadequate prenatal care and unwanted pregnancy [54]. Jansen et al. found no proportion eliminated for unplanned pregnancy [50].

### Composite measures

Meng et al. [39] assessed the proportion eliminated by three composite measures, estimating them as: 12% for SES-related support (maternal drug and alcohol abuse, single parent, financial difficulty, no prenatal care, no social support, maternal mental illness); 2% for psychosocial support (single parent, marital distress, family violence, smoking); and 6% for behaviour (infection, drug and alcohol abuse, single parent, financial difficulty, no prenatal care, family violence, smoking).

Misra et al. [41] found the proportion eliminated for health and stress was 64%. Räisänen et al. [43] found the proportion eliminated for smoking and other factors (placental abruption, placenta praevia, major congenital anomaly, anaemia, stillbirth, small for gestational age, and sex of infant) was 39% for extremely preterm births, 50% for very preterm births, and 41% for moderately preterm births.

Amegah et al. [45] found the proportion eliminated for malaria infection, pre-pregnancy BMI, and cooking fuel use combined was 30%. Morgen et al. [47] found the proportion eliminated for a combination of maternal behavioural mediators was 23% and 30% in primiparous and multiparous women, respectively. Niedhammer et al. [49] found the proportion eliminated for combined material, behavioural, and nutritional mediators was 42%. Jansen et al. [50] found the proportion eliminated for combined health, behavioural, and working patterns was 89% (Table 4).

Three studies found that inclusion of these composites removed statistical significance for the SES measures, which suggests complete mediation might be possible [41, 49, 50].

### Adjustment for confounders

Studies did not explicitly attribute confounders to the exposure-mediator, the mediator-outcome, or the exposure-outcome paths. The included studies considered various covariates for adjustment. Over three-quarters of the studies adjusted for maternal age as a confounder, and one study treated maternal age as a mediator. Parity was the next most frequently included covariate, included in over one-half of studies. Other notable covariates included ethnicity or race (both categorisations being used in different studies but referring to ethnic group), other measures of SES, and sex of the infant. Maternal health behaviours, health, stress, and prenatal care were all included in some studies as confounders, despite being examined as mediators in other studies. Multiple births and immigration status were more frequently used as exclusion criteria rather than confounders.

### Summary of mediation findings

**Fig. 3:**
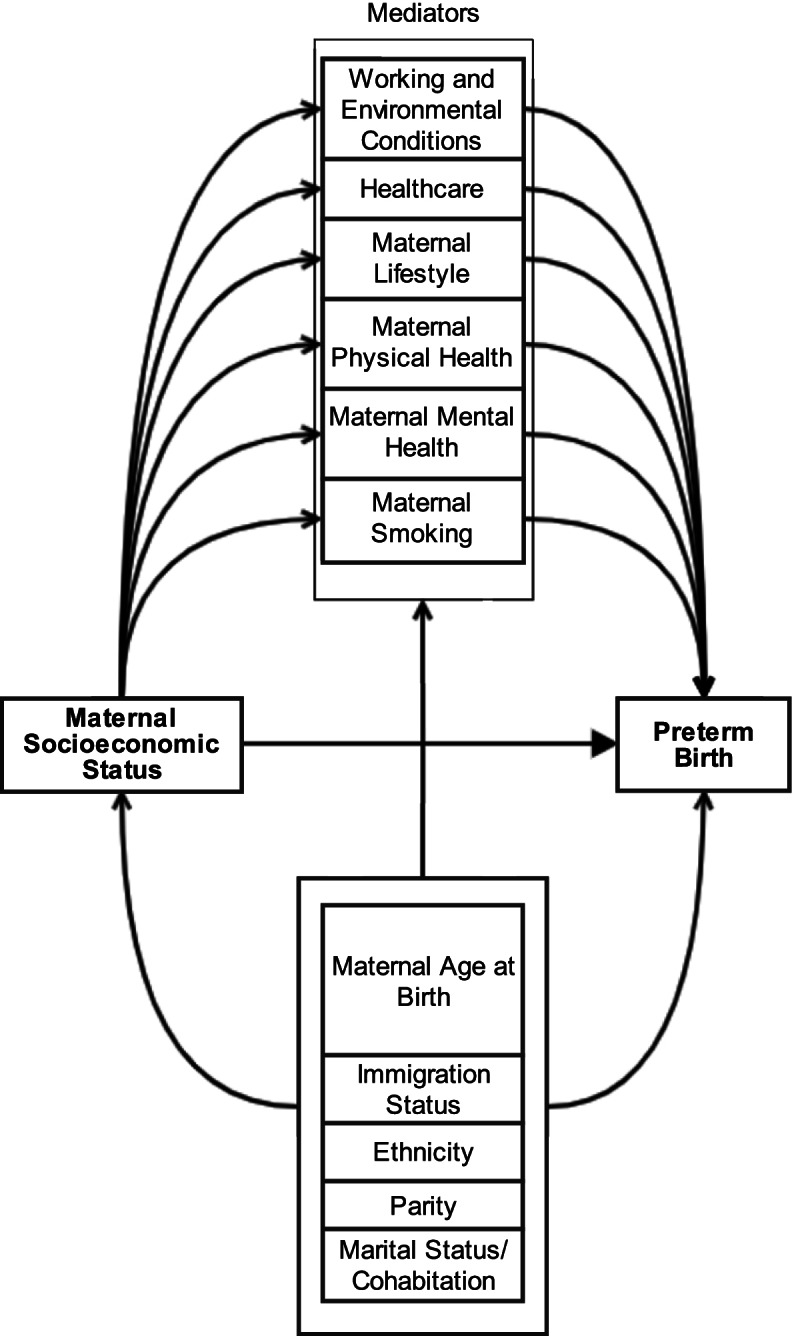
Causal pathway based on results of the studies included in the systematic review

The included studies analysed six groups of mediators (Fig. 3): maternal smoking; maternal mental health; maternal physical health (including BMI); maternal lifestyle (including alcohol consumption); healthcare; and working and environmental conditions.

Mediation through smoking was consistently demonstrated. Most studies did not calculate the CI of this, so it is not possible to assess precision. The studies that found small or non-significant effects tended to have smaller sample sizes while larger and higher quality studies found larger and statistically significant effects. There is high confidence of smoking being a mediator, however the size could not be estimated from this evidence.

There was mixed evidence that maternal mental health mediated the SES effect on preterm birth. The studies that found a significant indirect effect had the smallest sample sizes and highest quality, while the largest sample found no significant association between SES and preterm birth. The lowest quality study found no mediating effect. There is moderate confidence of maternal mental health being a mediator.

There is consistent evidence that there is significant mediation through maternal physical health, however the size of this effect depended on the way health was measured. Some specific conditions did not have a significant indirect effect. There is evidence of a significant indirect effect through pre-eclampsia, although this may differ by ethnicity. The evidence consistently shows that SES may have a small indirect effect through BMI, and one study found a significant indirect effect through BMI and gestational weight gain together. There is high confidence of maternal physical health being a mediator.

The evidence consistently shows that SES may have a small indirect effect through alcohol consumption. Despite the consistency, the lack of CI and the small effects mean there is low to moderate confidence that alcohol is a mediator. There is inconsistent evidence for working and environmental conditions, with no estimates of CI and only low-quality evidence for healthcare.

Confounders frequently used were maternal age, ethnicity or race, immigration status, parity, and marital status. It is important to note that the resulting path model (Fig. 3) is based on the evidence in this review and does not represent all variables and relationships that exist on this path or potential confounders.

## Discussion

### Principal findings

In aiming to identify evidence for mediation of the relationship between SES and preterm birth and to evaluate the quality of the methods used to assess mediation, this review finds that the current evidence is unable to answer our research question definitively. Mediation ranged from none to complete (the SES effect became non-significant), with no variable consistently mediating the effect of SES on preterm birth to the same extent across all studies.

Smoking was the most frequently examined mediator, with high confidence that smoking was a mediator of the effect of SES on preterm birth. There was also high confidence that maternal physical health was a mediator, however there was a wide range of measures of health, for example individual conditions and composite measures. There was lower confidence of mediation for the other identified variables being mediators. Most studies did not calculate the CI of the mediated effect; therefore, it is not possible to state confidently the size of this effect. The studies that found small or non-significant effects tended to have smaller sample sizes while larger and higher quality studies found larger and statistically significant effects.

Most included studies found a significant association between measures of SES and preterm birth prevalence, however the size of this effect ranged widely (from 6 to 185% increase in risk for low SES). Of the studies that found no significant effect of SES on preterm birth, two measured the effect of area-based SES while controlling for individual SES, risking overadjustment if area-based SES is taken as a proxy for individual SES. Two other studies measured the effect of area-based SES while controlling for individual SES, finding the effect significant.

Problems with the mediation methods affect our ability to make causal inferences. Most studies did not discuss the causal assumptions underpinning mediation. This is a particular issue for ‘cross-world independence’; a number of the mediators have inter-relationships, for example maternal health and health behaviours have an effect on obstetric complications [56, 57].

### Relevance to other studies

The effect of SES on birth outcomes has been well described, with a recent systematic review and meta-analysis showing significant associations between the wider social determinants of health and negative outcomes, including preterm birth [58]. Other studies, however, have shown a complicated relationship between mediators. Adhikari et al. demonstrated modification of the effect of depression and anxiety on preterm birth by SES [59]. McCall et al. found that, when stratified by smoking status, inequalities in preterm birth were only seen in non-smokers [60]. Studies have found mediation of inequalities in other perinatal outcomes (low birthweight, small for gestational age) [61, 62]. This adds support to the hypothesis that socioeconomic inequalities in preterm birth are at least partly explained by other exposures, however this relationship is potentially complicated by effect modification, highlighting the importance of incorporating exposure-mediator interaction into mediation analysis.

### Strengths of the study

The extensive searches of multiple databases, with supplementary searches, allow us to have high confidence that we have selected appropriate studies. Additionally, our quality appraisal included both biases associated with study design and quality of mediation approach. We included all study designs and measures of SES to maximise the evidence available to us for the review.

### Limitations of the review

Our inclusion criteria meant there are two major limitations. First, different measures of SES are potentially not comparable. The measure of SES used will affect the extent of inequalities observed in preterm birth [63, 64], particularly when considering area-based and individual measures [65, 66]. There is evidence that disagreement can occur between these measures [67], suggesting that the pathways to inequalities may differ. Notably though, our study showed no clear differences based on measure of SES used, therefore we are considering the different measures as broadly comparable exposures.

Second, only eight studies made clear that the exposure was measured before the outcome, yet temporality is a requirement for causal interpretation. Nevertheless, SES could be argued to be a relatively static exposure in the perinatal period (depending on measurement) so the importance of this potential problem is debatable.

Finally, our search strategy focused on studies that explicitly examined mediation or explanation of inequalities in preterm birth. This could potentially lead to missing studies in which a mediated effect could still be extracted. If the aim of the study was not to assess mediation, however, the causal relationships and pathways would not have been considered. Such an estimation would not have considered confounding, leading to flawed estimates. Minor deviations from the PROSPERO protocol were noted, however these have not impacted on our findings or introduced a new risk of bias.

### Limitations of the data

Of limitations in the evidence, first, some potential mediators were not examined. For example, air pollution [68–70], urbanicity [71], and domestic violence [72] have been shown to affect preterm birth risk and are socially patterned and thus are plausible mediators of preterm birth inequalities. Particularly relevant is that the focus of included mediators tends to be individual (behaviours, health status) rather than more upstream and systems-based variables such as access to healthcare and other determinants. Second, assessing the measurement of the included mediators was problematic. For example, some mediators were not measured during pregnancy and were aggregated [48], and some composites combined seemingly unconnected mediators [39, 43].

Third, most studies treated preterm birth as a homogenous group, however extremely preterm birth and late preterm birth differ in both consequences and causes [73]. Most studies did not report whether the preterm birth was iatrogenic or spontaneous, which affects risks of adverse consequences, however the link with SES is unclear [74, 75]. Fourth, most of the included studies did not estimate CI for the proportion eliminated and 11 studies did not estimate mediator significance (all used the difference method) [76], limiting our synthesis. This means that studies including the same mediators do not necessarily show different results but differences found may be due to uncertainty in the effect that we were unable to quantify. Finally, not assessing the exposure-mediator interaction can significantly and substantially bias results.

## Conclusions

Effective intervention to reduce inequalities in preterm birth may involve action on mediators of the effect of maternal SES on preterm birth. Complete mediation of the SES effect on preterm birth is unlikely by individual variables, given that most studies show a large residual direct effect of SES. This suggests that a focus on modifiable socioeconomic determinants, such as reducing poverty and educational inequality, is necessary to address inequalities in preterm birth, alongside action on mediating pathways.

Given the variable quality of the evidence, from the study design and particularly the mediation methods used, there is a pressing need for more robust primary research into mediation to identify causal evidence to inform policy. The evidence does suggest that risk factors lying on the pathway from SES to preterm birth explain some of the inequalities in preterm birth. Action on smoking is most strongly supported, for example through financial incentives [77]. Overall though, the current evidence precludes ranking these risks to maximise outcomes from policy action.

## Supplementary Information

**Additional file 1:**

**Additional file 2:** Deviations from PROSPERO protocol

**Additional file 3:** Search Strategy

**Additional file 4:** QualityAppraisal

**Additional file 5: **Table showing quality appraisal score for each study; *Conf. *Confounding, *Int. *Interaction, *Assump. *Assumptions

## Data Availability

All data generated or analysed during this study are included in this published article and its supplementary information files.

## Abbreviations

BMI: Body mass index
DAG: Directed acyclic graph
SES: Socioeconomic status
