# Supplementary material for: Mediators of socioeconomic inequalities in preterm birth: a systematic review
Source: BMC Public Health. 2022 Jun 7;22:1134. doi: 10.1186/s12889-022-13438-9 (PMC9172189; doi:10.1186/s12889-022-13438-9)
Supplement: Supplementary file 2 — Additional file 2: Deviations from PROSPERO protocol [file 12889_2022_13438_MOESM2_ESM.docx]

# Appendix B – Deviations from PROSPERO protocol

There were three minor deviations from our protocol:

- We double-screened 15% of titles and abstracts rather than 20%. When we calibrated at 15%, agreement was high between the two reviewers, and further double-screening was not required.
- The search strategy was expanded and further detailed in the manuscript. A detailed search strategy was not included in the protocol.
- The Liverpool Quality Assessment tool was modified to appraise quality in the context of mediation. We used this rather than the Newcastle-Ottawa tool as it covered the areas we identified in the protocol plus mediation quality in a straightforward way.

These minor changes have not impacted on our findings or introduced a new risk of bias.
