# Supplementary material for: Mediators of socioeconomic inequalities in preterm birth: a systematic review
Source: BMC Public Health. 2022 Jun 7;22:1134. doi: 10.1186/s12889-022-13438-9 (PMC9172189; doi:10.1186/s12889-022-13438-9)
Supplement: Supplementary file 3 — Additional file 3: Search Strategy [file 12889_2022_13438_MOESM3_ESM.docx]

# Appendix C – Search Strategy

The included search terms were:

1. mediat* OR (structural equation modelling) OR (structural equation modelling) OR path OR (Baron and Kenny) OR MacKinnon OR (product of coefficient) OR (difference in coefficient) OR (process of change) OR sobel* OR (causal pathway) OR intermediate OR (process variable) OR (treatment ADJ2 effect) OR (process ADJ2 evaluation) OR mechanism OR SEM OR modifi* or interact* or (differential exposure) or (differential susceptibility) or attenuat* or indirect* or decomp*
2. disparit* OR socio-economic OR socioeconomic OR depriv* OR inequal* OR poverty OR inequit* OR disadvantag*
3. preterm OR prematur* OR gestation
4. 1 AND 2 AND 3

Search terms were consistent across all databases. Only the operators varied, as required for each database. The same search terms were used for Advanced Google search, however limitations in number of terms required the search to split into five sub-searches, as follows:

- (“mediat*” | “structural equation modelling” | “path” | “attenuat*” | “indirect*” | “decomp*”) (“disparit*” | “socioeconomic” | “depriv*” | “inequal*” | “poverty” | “inequit*” | “disadvantag*”) (“preterm” | “prematur*” | “gestation*”)
- (“Baron and Kenny” | “MacKinnon” | “product of coefficient” | “difference in coefficient” ) (“disparit*” | “socioeconomic” | “depriv*” | “inequal*” | “poverty” | “inequit*” | “disadvantag*”) (“preterm” | “prematur*” | “gestation*”)
- (“process of change” | “sobel*” | “causal pathway” | “intermediate” | “process variable”) (“disparit*” | “socioeconomic” | “depriv*” | “inequal*” | “poverty” | “inequit*” | “disadvantag*”) (“preterm” | “prematur*” | “gestation*”)
- (“treatment ADJ2” | “process ADJ2 evaluation” | “mechanism” | “SEM” | “modifi*” | “interact*”) (“disparit*” | “socioeconomic” | “depriv*” | “inequal*” | “poverty” | “inequit*” | “disadvantag*”) (“preterm” | “prematur*” | “gestation*”)
- (“differential exposure” | “differential susceptibility”) (“disparit*” | “socioeconomic” | “depriv*” | “inequal*” | “poverty” | “inequit*” | “disadvantag*”) (“preterm” | “prematur*” | “gestation*”)

The first ten pages for each search were screened.
