# Supplementary material for: Mediators of socioeconomic inequalities in preterm birth: a systematic review
Source: BMC Public Health. 2022 Jun 7;22:1134. doi: 10.1186/s12889-022-13438-9 (PMC9172189; doi:10.1186/s12889-022-13438-9)
Supplement: Supplementary file 4 — Additional file 4: QualityAppraisal [file 12889_2022_13438_MOESM4_ESM.docx]

# Appendix D – Quality Appraisal

Selection

- Cohort: selection bias, response bias, follow-up bias
- Cross-Sectional: selection bias, response bias
- Case Control: selection of cases, selection of controls

Measurement

- Cohort: SES measure (individual or aggregate), measurement bias for mediators/preterm birth, ascertainment bias of preterm birth
- Cross-Sectional: SES measure (individual or aggregate), measurement bias for mediators/preterm birth, recall bias for exposure/mediators, ascertainment bias of preterm birth
- Case Control: SES measure (individual or aggregate), measurement bias for mediators/preterm birth, recall bias for exposure/mediators, ascertainment bias of preterm birth

Confounding

- Control for ethnicity/race, maternal age, parity

Mediation

- DAG included
- Exposure-mediator interaction in analysis
- Explicit consideration of causal assumptions
