# Supplementary material for: Mediators of socioeconomic inequalities in preterm birth: a systematic review
Source: BMC Public Health. 2022 Jun 7;22:1134. doi: 10.1186/s12889-022-13438-9 (PMC9172189; doi:10.1186/s12889-022-13438-9)
Supplement: Supplementary file 5 — Additional file 5: Table showing quality appraisal score for each study; Conf. Confounding, Int. Interaction, Assump. Assumptions [file 12889_2022_13438_MOESM5_ESM.docx]

**Appendix E – Table showing quality appraisal score for each study; Conf.=Confounding, Int.=Interaction, Assump.=Assumptions**

| **Author** | **Selection bias** | **Response bias** | **Selection Cases** | **Selection Controls** | **Bias follow-up** | **SES** | **Recall exposure and mediators** | **Measurement bias mediators** | **Ascertainment bias** | **Measurement bias PTB** | **Conf.** | **Graph** | **Int.** | **Causal Assump.** | **Total Score** |
| --- | --- | --- | --- | --- | --- | --- | --- | --- | --- | --- | --- | --- | --- | --- | --- |
| Poulsen *et al.* (2019) | 1 | 1 | n/a | n/a | 0 | 2 | n/a | 0 | 1 | 1 | 1 | 1 | 1 | 1 | 10 |
| Ross *et al.* (2019) | 0 | 1 | n/a | n/a | 1 | 2 | n/a | 1 | 1 | 0 | 2 | 1 | 0 | 0 | 9 |
| Dolatian *et al.* (2014) | 1 | 0 | n/a | n/a | 1 | 2 | n/a | 1 | 1 | 1 | 1 | 1 | 0 | 0 | 9 |
| Clayborne *et al.* (2017) | 0 | 1 | n/a | n/a | 1 | 1 | n/a | 1 | 1 | 0 | 2 | 1 | 0 | 0 | 8 |
| Dooley (2009) | 1 | 0 | n/a | n/a | n/a | 1 | 1 | 0 | 1 | 1 | 1 | 1 | 1 | 0 | 8 |
| Mehra *et al.* (2019) | 0 | 1 | n/a | n/a | 1 | 1 | n/a | 1 | 1 | 0 | 1 | 1 | 1 | 0 | 8 |
| Meng *et al.* (2013) | 1 | 0 | n/a | n/a | n/a | 1 | 1 | 0 | 1 | 0 | 1 | 1 | 1 | 1 | 8 |
| Mirabzadeh *et al.* (2013) | 1 | 0 | n/a | n/a | 1 | 2 | n/a | 1 | 1 | 1 | 0 | 1 | 0 | 0 | 8 |
| Misra *et al.* (2001) | 1 | 0 | n/a | n/a | n/a | 2 | 0 | 1 | 1 | 1 | 1 | 1 | 0 | 0 | 8 |
| Räisänen *et al.* (2013) | 1 | 1 | n/a | n/a | n/a | 2 | 1 | 0 | 1 | 1 | 1 | 0 | 0 | 0 | 8 |
| Nkansah-Amankra *et al.* (2010) | 1 | 1 | n/a | n/a | n/a | 1 | 1 | 0 | 1 | 1 | 1 | 0 | 1 | 0 | 8 |
| Amegah *et al.* (2013) | 1 | 0 | n/a | n/a | n/a | 2 | 1 | 0 | 1 | 1 | 1 | 0 | 0 | 0 | 7 |
| Morgen *et al.* (2008) | 1 | 1 | n/a | n/a | 0 | 2 | n/a | 0 | 1 | 1 | 1 | 0 | 0 | 0 | 7 |
| van den Berg *et al.* (2012) | 0 | 1 | n/a | n/a | 1 | 2 | n/a | 0 | 1 | 1 | 1 | 0 | 0 | 0 | 7 |
| Niedhammer *et al.* (2012) | 1 | 0 | n/a | n/a | 1 | 2 | n/a | 0 | 1 | 1 | 1 | 0 | 0 | 0 | 7 |
| Gisselmann and Hemström (2008) | 1 | 1 | n/a | n/a | n/a | 2 | 1 | 0 | 1 | 0 | 1 | 0 | 0 | 0 | 7 |
| Jansen *et al.* (2009) | 1 | 0 | n/a | n/a | 1 | 2 | n/a | 0 | 1 | 1 | 1 | 0 | 0 | 0 | 7 |
| Ahern *et al.* (2003) | n/a | n/a | 1 | 1 | n/a | 1 | 1 | 0 | 1 | 0 | 1 | 0 | 1 | 0 | 7 |
| Quispel *et al.* (2014) | 1 | 0 | n/a | n/a | 0 | 2 | n/a | 1 | 1 | 0 | 0 | 1 | 0 | 0 | 6 |
| Gissler *et al.* (2003) | 0 | 1 | n/a | n/a | n/a | 2 | 1 | 0 | 1 | 0 | 1 | 0 | 0 | 0 | 6 |
| Gray *et al.* (2008) | 1 | 0 | 1 | n/a | n/a | 1 | n/a | 0 | 1 | 1 | 1 | 0 | 0 | 0 | 6 |
| de Oliveira *et al.* (2019)^54^ | n/a | n/a | 0 | 0 | n/a | 2 | 0 | 1 | 0 | 0 | 0 | 1 | 0 | 0 | 4 |
